# Prevalence and care-seeking for chronic diseases among Syrian refugees in Jordan

**DOI:** 10.1186/s12889-015-2429-3

**Published:** 2015-10-31

**Authors:** Shannon Doocy, Emily Lyles, Timothy Roberton, Laila Akhu-Zaheya, Arwa Oweis, Gilbert Burnham

**Affiliations:** Johns Hopkins Bloomberg School of Public Health, 615 N Wolfe St, Suite E8132, Baltimore, MD 21205 USA; School of Nursing, Jordan University of Science and Technology, Irbid, Jordan

**Keywords:** Syria, Jordan, Refugees, Humanitarian assistance, Non-communicable diseases, Care-seeking, Prevalence

## Abstract

**Background:**

There are currently more people displaced by conflict than at any time since World War II. The profile of displaced populations has evolved with displacement increasingly occurring in urban and middle-income settings. Consequently, an epidemiological shift away from communicable diseases that have historically characterized refugee populations has occurred. The high prevalence of non-communicable diseases (NCDs) poses a challenge to in terms of provision of appropriate secondary and tertiary services, continuity of care, access to medications, and costs. In light of the increasing burden of NCDs faced by refugees, we undertook this study to characterize the prevalence of NCDs and better understand issues related to care-seeking for NCDs among Syrian refugees in non-camp settings in Jordan.

**Methods:**

A cross-sectional survey of 1550 refugees was conducted using a multi-stage cluster design with probability proportional to size sampling to obtain a nationally representative sample of Syrian refugees outside of camps. To obtain information on chronic conditions, respondents were asked a series of questions about hypertension, cardiovascular disease, diabetes, chronic respiratory disease, and arthritis. Differences by care-seeking for these conditions were examined using chi-square and *t*-test methods and characteristics of interest were included in the adjusted logistic regression model.

**Results:**

Among adults, hypertension prevalence was the highest (9.7 %, CI: 8.8–10.6), followed by arthritis (6.8 %, CI: 5.9–7.6), diabetes (5.3 %, CI: 4.6–6.0), chronic respiratory diseases (3.1 %, CI: 2.4–3.8), and cardiovascular disease (3.7 %, CI: 3.2, 4.3). Of the 1363 NCD cases, 84.7 % (CI: 81.6–87.3) received care in Jordan; of the five NCDs assessed, arthritis cases had the lowest rates of care seeking at 65 %, (CI:0–88, *p* = 0.005). Individuals from households in which the head completed post-secondary and primary education, respectively, had 89 % (CI: 22–98) and 88 % (CI: 13–98) lower odds of seeking care than those with no education (*p* = 0.028 and *p* = 0.037, respectively). Refugees in North Jordan were most likely to seek care for their condition; refugees in Central Jordan had 68 % (CI: 1–90) lower odds of care-seeking than those in the North (*p* = 0.047).

**Conclusion:**

More than half of Syrian refugee households in Jordan reported a member with a NCD. A significant minority did not receive care, citing cost as the primary barrier. As funding limitations persist, identifying the means to maintain and improve access to NCD care for Syrian refugees in Jordan is essential.

## Background

Over the past two decades, crises like those in Haiti, Iraq, Somalia, and Yemen have brought the phenomenon of displacement in urban settings into prominence. The majority of the world’s refugees now live in urban areas. As the number of refugees has increased there have also been shifts towards middle-income settings with older post-demographic transition structures and an epidemiological shift away from communicable diseases that have historically characterized refugee populations [1, 2]. Traditional camp-based assistance models have been adapted to better support refugees in urban environments; in 2009, UNHCR adapted its policy on refugees in urban settings to involve integration of refugees into existing host country systems for health, education, and other basic needs [3, 4].

While traditional priorities in humanitarian assistance remain, the burden of non-communicable diseases (NCDs) in displaced populations is increasing [5]. The high prevalence of NCDs in many refugee and host country populations pose challenges to providing appropriate secondary and tertiary services, continuity of care, access to medications, and costs. At the health system level, NCDs require more sophisticated diagnostic and management capacity than many communicable diseases. Limited awareness, access to diagnostic testing, and inadequate treatment for NCDs can lead to delayed care-seeking, complications requiring sophisticated treatments, and preventable adverse health outcomes [6].

Since the start of unrest in March 2011, the Syrian conflict has caused an estimated 4.6 million Syrians to flee the country [7]. As of May 2015, more than 3.97 million Syrian refugees were registered or awaiting registration with UNHCR, in addition to an unregistered population that is unknown in size [8]. Jordan alone is host to over 627,000 registered Syrian refugees, the vast majority of who reside in Jordanian communities along the northern border governorates (Irbid and Mafraq) and in greater Amman rather than in formal camps [9]. With little or no development of parallel systems to serve refugees in host countries, the burden on host country health systems, including demand for surgery, trauma care, maternal health, and chronic disease care, has increased dramatically as a result of the influx of Syrian refugees [10–12]. At the time of data collection, Syrian refugees registered with UNHCR in Jordan were able to access primary and secondary health care free of charge at Public Health Centers and, when needed, Governmental Hospitals (with referral from Public Health Centers); however, the cost of providing uninterrupted care for chronic health conditions remains costly for UNHCR, implementing agencies, and the Government of Jordan [13]. Despite the Jordanian health system’s progress preceding the Syrian crisis, the rapid influx of refugees is projected to result in a reversal in the country’s progress in many key Millennium Development Goal indicators [14]. The immense burden placed on the Jordanian health system lead to revised policies in November 2014 in which Syrian refugees are now required to pay the same rate for care at Public Health Care facilities as uninsured Jordanians [15].

Cost is not the only challenge the international community faces in meeting refugee health needs. Long wait times, poor staff treatment, and transportation also often prevent care-seeking, exhibiting the increasing strain on Jordan’s capacity and infrastructure in health in addition to a number of other sectors [15–18]. Additionally, many refugees are not seeking care because they do not know where to go or do not think they are sick enough to require the effort and cost associated with seeking care, underscoring the need for improved education campaigns to inform refugees of the resources available to them and when care is necessary [17]. Perceived inequality in access to health care by both refugees and host communities stemming from overcrowding of the health sector and uneven access between Jordanians and Syrians both contribute to reduced social cohesion and social resilience in the Jordanian population [14]. With no end to the Syrian crisis in sight the burdens faced by refugee host countries will likely increase in the coming years. In light of this, we undertook this study to characterize the prevalence of NCDs and better understand issues related to care-seeking for NCDs among Syrian refugees in non-camp settings in Jordan.

## Methods

A cross-sectional survey of Syrian refugees in Jordan was conducted in June 2014 to characterize prevalence of NCDs and better understand issues related to NCD care-seeking. A multi-stage cluster survey design with probability proportional to size sampling was used to obtain a nationally representative sample of Syrian refugees living outside of camps. Sample size was determined for key study objectives based on the most conservative prevalence rate estimate of 50 %; calculations assumed 80 % power and a design effect of 2.0 to account for the cluster sample design. The planned sample size was increased to compensate for a non-response rate of up to 10 %. The planned sample size was increased from the minimum identified size of 900 households to 1500 households to provide increased precision of point estimates and additional power for the detection of statistically significant differences of >10 % for the sub-national comparison of key indicators.

**Fig. 1:**
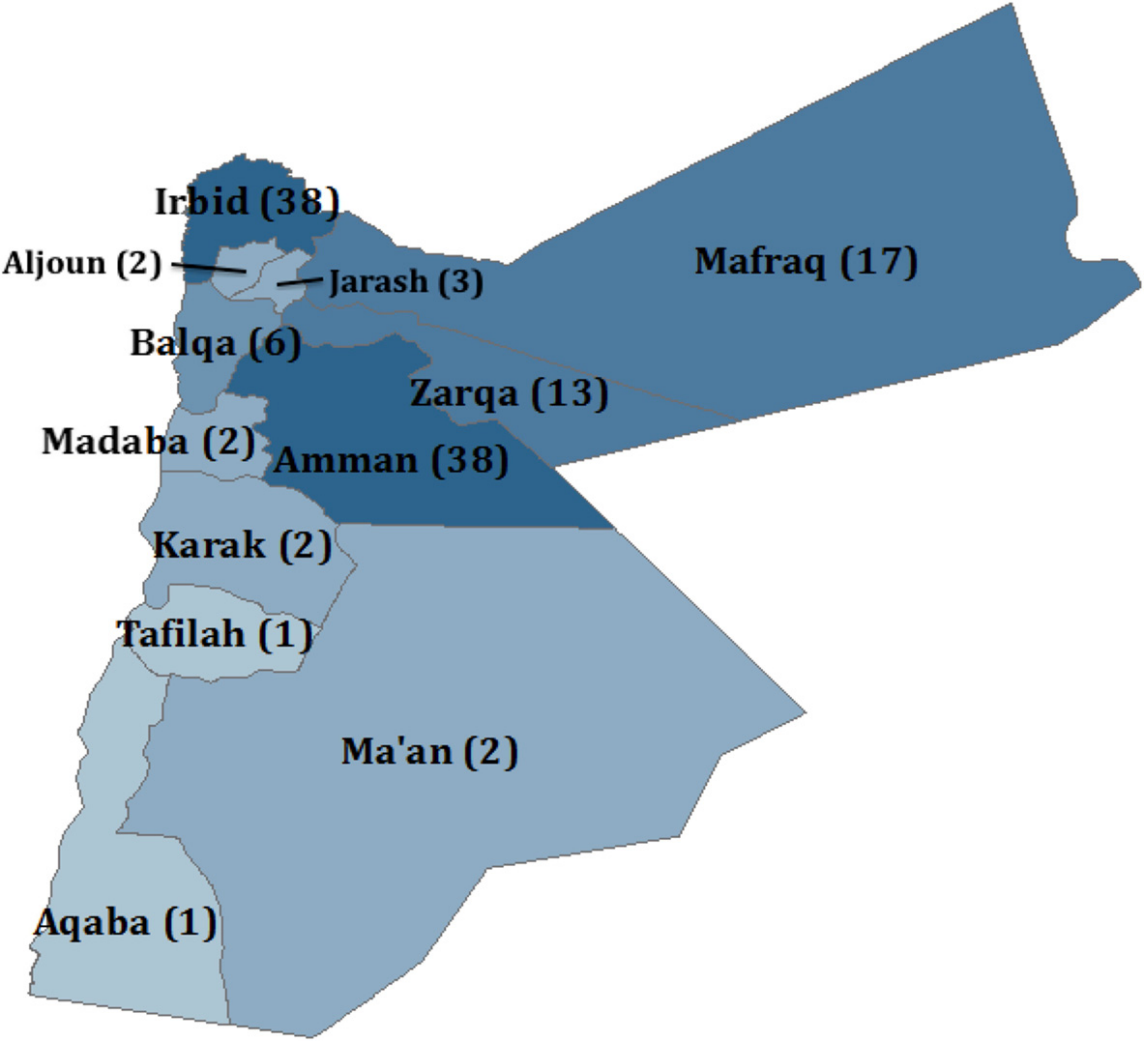
Distribution of study clusters

Given the localization of Syrian refugees and the low cost of visiting many locations in Jordan due to the country’s small size and good transportation infrastructure, a 125 cluster x 12 household design was chosen. Probability proportional to size sampling was used to assign the number of clusters to sub-districts using UNHCR registration data, assuming that non-registered refugees had similar residence patterns. The final cluster assignment included 38 clusters (30 %) in Amman governorate, 38 clusters (30 %) in Irbid governorate, and 49 clusters (40 %) distributed proportionately in the remaining governorates (Fig. 1). The UNHCR Amman office randomly selected five registered refugee households that were listed as living in that cluster’s assigned sub-district as a cluster start point. The study team then called households; the first household currently residing in the specified sub-district that agreed to meet with the study team was used as the starting household for the cluster. The study team met this household, conducted an abbreviated interview (the results of which were not included in the survey data set to minimize bias towards registered refugees), and enquired about the nearest Syrian household. The household(s) to which the starting household referred the interview teams were then interviewed using the complete questionnaire. Respondents were most often household heads or caretakers of and they answered questions on behalf of the households and its members; where possible other household members participated and answered specific relevant questions. Household members were defined as people who share a dwelling space and share meals, regardless of biological relation; short-term visitors were not counted as household members. At the conclusion of each interview, households were asked for a referral to the nearest Syrian household. This was continued until twelve interviews were completed in a cluster. Only Syrian households arriving in Jordan in 2011 or after were eligible to participate in the survey; however, none of the households approached for interview arrived in Jordan before 2011. To ensure that the nearest referred Syrian households were refugees, a question was asked at the start of the interview about the year of arrival in Jordan. If respondents arrived in 2010 or earlier, interviewers were prompted to the end of the questionnaire and ended the interview by thanking respondents and explaining the reason for exclusion of non-refugee Syrians.

To obtain information on access to health services and medications for chronic conditions, respondents were asked about the five conditions reported to be most common among the Syrian refugee population in Jordan: hypertension, cardiovascular disease,^1^ diabetes, chronic respiratory disease,^2^ and arthritis [17, 19]. Diagnosis was self-reported; no documentation was required to verify the conditions. Respondents were asked a series of questions about each of these conditions, beginning with the number of people in the household diagnosed with the condition organized into four age groups: under 17 years, 18–39 years, 40–59 years, and 60 years and over. A series of questions regarding care-seeking, access, and utilization of health services were asked about the experiences of one randomly selected household member who had been diagnosed with the each of the conditions. If more than one person in the household had that condition, the person whose birthday was closest to the date of the interview was asked the series of questions about care-seeking. Household economic status was characterized by both income and expenditures. Expenditures were measured as a total of reported spending in a number of categories (health, food, housing, education, etc.) in the month preceding the survey. Income included the value of income in the month preceding the survey from a variety of sources such as salaries, pensions, investments, or remittances, but excluded cash or vouchers received as humanitarian assistance. Average monthly expenditures are believed to more likely reflect household wealth, notably available savings, and be a more valid measure of socioeconomic status in this context due to difficulties associated with accurately collecting income data as a result of sensitivities related to refugees not being able to work legally and misconceptions that households reporting low income will be targeted for humanitarian assistance.

The questionnaire was developed following discussions between UNHCR, WHO, JHU, and stakeholder consultations. By consensus, the focus of the questionnaire was on health service utilization, access to care, barriers to care-seeking, children’s health and vaccination, and chronic medical conditions. The questionnaire was translated to Arabic and both a pre-pilot test and a formal pilot test were performed. Interviews were conducted by Jordanian interviewers that underwent two days of classroom training on the questionnaire, e-data collection using tablets, interview techniques, basic principles of human subjects’ protections and sampling methods followed by one day of practical field training. To protect the anonymity of respondents, no information was recorded that could be used to identify the household or individuals and verbal consent was obtained from all respondents. This point, along with the fact that participation would not influence any assistance received, was stressed when approaching households to mitigate unregistered refugees’ fears of participation. Interviews lasted between 30 and 60 min depending on the household size, number of children and individuals with chronic medical conditions.

Data was collected on tablets using the Magpi mobile data platform by DataDyne LLC (Washington, DC). Data was analyzed using Stata 13 (College Station, TX) and Tableau Desktop (Seattle, WA) software packages and employed standard descriptive statistics and methods for comparison of means and proportions. Differences in household characteristics by care-seeking in Jordan were examined using chi-square and *t*-test methods. The Stata ‘*svy’* command was used to account for the cluster survey design so that standard errors of the point estimates and model coefficients were adjusted for survey design effects.

The study was reviewed and approved by ethics committees at the World Health Organization, Jordan University of Science and Technology and Johns Hopkins School of Public Health and the Jordanian Ministry of Health.

## Results

### Background characteristics

**Fig. 2:**
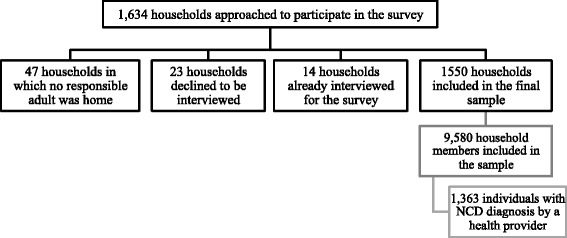
Participant flow chart

A total of 1634 households were approached to participate in the survey. Of these households, in 2.9 % (*n* = 47) no responsible adult was at home, 0.8 % (*n*= 14) were already interviewed for this survey, and 1.4 % (*n* = 23) declined to be interviewed (Fig. 2). The final sample included 1550 households (with 9580 household members), which equates to a response rate of 94.7 %. Educational attainment among household heads and respondents was low, with less than a quarter having completed secondary school. Of the households included in the survey, 43.9 % arrived in 2011 and 2012; the remaining 56.1 % arrived in 2013 and within the first six months of 2014. A substantial portion (94.8 %) of households reported registration of all members with UNHCR. Crowding, defined as the number of household members per sleeping room, was assessed as a potential proxy for socioeconomic status and living conditions. Based on the distribution, five or more people per sleeping room was the established cutoff for crowding and 13.2 % (CI: 10.7–16.3) of households fell within this category. Among all households, the mean monthly expenditure was 538 USD (CI: 513–563; median = 474). Median monthly expenditures in the top quartile were 792 USD as compared to 285 USD in the lowest quartile.

### Prevalence of chronic health conditions

Of the 1550 households surveyed, 780 (50.3 %, CI: 47.2-53.4) reported one or more household members with previous diagnosis by a health provider with one of the five non-communicable diseases included in the survey (hypertension, cardiovascular disease, diabetes, chronic respiratory diseases, and arthritis).^3^ A total of 1363 individual NCD cases reported on care seeking, where for each condition, one individual in the household was selected for reporting, but multiple NCD cases could be reported in a household if they were for different conditions.

**Fig. 3:**
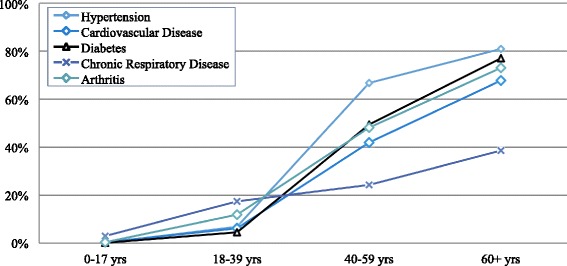
Age specific prevalence rates of chronic health conditions

Among adult household members (≥18 years), reported hypertension prevalence was the highest (10.7 %, CI: 9.8–11.7), followed by arthritis (7.1 %, CI: 6.2–7.9), diabetes (6.1 %, CI: 5.5–6.8), cardiovascular disease (4.1 %, CI: 3.5, 4.7), and chronic respiratory diseases (2.9 %, CI: 2.3–3.4). Arthritis (2.5 %, CI: 1.9–3.1) and chronic respiratory disease (2.2 %, CI: 1.6–2.7) were the most prevalent chronic conditions reported in the 18 to 39 year old population. Prevalence of all reported non-communicable disease conditions was substantially higher in those 40 years old and over (Table 1 and Fig. 3). Hypertension was most prevalent reported condition in the 40–59 and 60+ age categories, with prevalence rates of 21.1 % (CI: 18.6–23.7) and 52.1 % (CI: 46.5–57.7), respectively. None of the five non-communicable diseases were commonly reported among children; for 0–17 year olds, the most prevalent reported chronic condition was chronic respiratory diseases (3.0 %, CI: 2.1–3.8). Because no identifying information was collected from participants, it was not possible to identify individuals with more than one NCD and, as such, double burden of NCDs in individual household members was not assessed.

**Table 1:** Age-specific chronic disease prevalence

	Survey Total N	Hypertension (*N* = 3277)		Cardiovascular Disease (*N* = 1289)		Diabetes (*N* = 1970)		Chronic Respiratory Disease (*N* = 790)		Arthritis (*N* = 2187)	
		N	% (95 % CI)	N	% (95 % CI)	N	% (95 % CI)	N	% (95 % CI)	N	% (95 % CI)
Households where any member(s) have condition	1550	408	26.3 (24.0–28.8)	190	12.3 (10.6–14.2)	250	16.1 (14.4–18.0)	213	13.7 (12.0–15.7)	302	19.5 (17.3–21.9)
Prevalence by Age Group											
0–17 years	5147	6	0.1 (0–0.2)	20	0.4 (0.1–0.7)	8	0.2 (0.0–0.3)	153	3.0 (2.1–3.8)	18	0.3 (0.1–0.6)
18–39 years	3019	60	2.0 (1.4–2.6)	25	0.8 (0.5–1.2)	26	0.9 (0.5–1.2)	66	2.2 (1.6–2.7)	75	2.5 (1.9–3.1)
40–59 years	1040	220	21.1 (18.6–23.7)	78	7.5 (5.9–9.2)	125	12.0 (10.0–14.0)	39	3.8 (2.5–5.0)	154	14.8 (12.5–17.1)
60+ years	374	195	52.1 (46.5–57.7)	80	21.4 (17.0–25.8)	121	32.4 (27.3–37.4)	22	5.9 (3.2–8.6)	85	23.7 (18.1–27.3)
Adult Prevalence^a^	4433	475	10.7 (9.8–11.7)	183	4.1 (3.5–4.7)	272	6.1 (5.5–6.8)	127	2.9 (2.3–3.4)	314	7.1 (6.2–7.9)

^a^“Adult” defined as individual over 17 years old

### Care-seeking for chronic health conditions

Care-seeking for chronic health conditions was high across all conditions. Of the 1363 non-communicable disease cases, 84.7 % (CI: 81.6–87.3) reported receiving care in Jordan. The last time careseekers received medical care for each condition was as follows: <1 month ago, 46.7 % (CI: 42.8–50.7); 1 to less than 3 months ago, 22.8 % (CI: 20.2–25.7); 3 to less than 6 months ago, 8.9 % (CI: 7.3–10.7); 6 months to less than one year ago, 3.3 % (CI: 2.2–5.0) and more than one year ago, 2.9 % (CI: 2.0-4.4). Significant differences in care-seeking were observed among regions with highest rates of care-seeking in the North (89.0 % CI: 85.3–91.8) and the lowest in the Central region (79.5 %, CI: 74.7–83.6) (*p* = 0.004).

NCD care was often received recently among respondents with more than half of cases in all conditions reporting receiving care in the past three months. The largest proportion of cases receiving care in the past 3 months was among hypertension cases (77.7 %, CI: 73.3–81.6) and the lowest among arthritis cases (58.3 %, CI: 52.3–64.0). The proportion of cases that reported seeing a doctor in Jordan for the condition was high among all NCDs with the highest proportion observed in those with chronic respiratory disease (87.8 %, CI: 83.1–91.3) and the lowest in arthritis cases (75.8 %, CI: 69.8–81.0). No significant regional difference in care-seeking was observed among cardiovascular disease (*p* = 0.447) and arthritis cases (*p* = 0.174). Regional differences in care-seeking were reported by cases of hypertension with a higher percentage of those in the South with hypertension seeking care (94.1 %, CI: 69.7–99.1) and the lowest care-seeking percentage in the Central Region (83.3 %, CI: 76.7–88.3) (*p* = 0.037). Significant differences in care-seeking were also observed in reported diabetics and chronic respiratory disease cases among whom a higher percentage of cases sought care in the North (93.4 % of diabetics, CI: 88.0–96.5; 91.8 % of chronic respiratory disease cases, CI: 86.0–95.3) and a lower percentage in the Central Region (81.2 %, CI: 71.9–87.9 of diabetics; 81.5 %, CI: 72.6–68.0 of chronic respiratory disease cases) (*p* = 0.044 and 0.031, respectively). Inability to afford care was the most common reason for not seeking care in each of the included conditions.

**Fig. 4:**
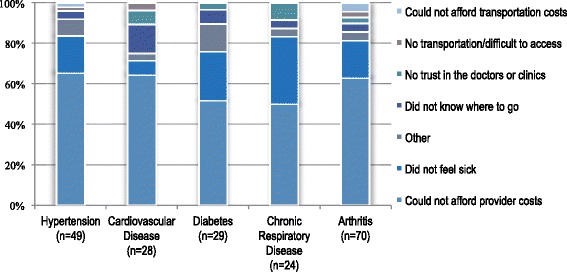
Reasons for not seeking care for chronic conditions

The proportions of non-care seekers citing cost as the reason for not seeking care are as follows: hypertension, 65.3 % (CI: 61.0–77.3); cardiovascular disease, 64.3 % (CI: 44.7–80.1); diabetes, 51.7 % (CI: 33.9–69.2); chronic respiratory disease, 50 % (CI: 30.8–69.2); arthritis, 62.9 % (CI: 51.8–72.7). Additional reasons were not as uniformly reported in all conditions. Individuals with hypertension (4.1 %, CI: 1.0–14.9) and cardiovascular disease (14.3 %, CI: 5.3–33.3) also reported not seeking care because they did not know where to go. Additionally, some cases reported not feeling sick enough to seek care for all NCDs except cardiovascular disease (Fig. 4).

#### Predictors of care-seeking

Results of univariate and multivariate logistic regression analyses for predictors of care-seeking for chronic health conditions among Syrian refugees in Jordan are presented in Table 2. Household head education status, geographic region of residence in Jordan, and specific health condition reported were significantly associated with odds of seeking care in Jordan in both univariate and multivariate regression analyses. Receipt of non-food item assistance was significantly associated with odds of care-seeking in multivariate analysis, but not in univariate analysis. There was an inverse relationship between care-seeking and household head educational attainment: households in which the head had completed more than secondary education had 89 % (CI: 22–98) lower odds of seek care compared to those with no education and households in which the head completed primary school had 88 % (CI: 13–98) lower odds of seeking care than those with no education; no significant differences in care-seeking rates were observed between households in which the head completed preparatory education and with no education completed. Additionally, households in Central Jordan had 68 % (CI: 1–90) lower odds of care-seeking than those in North Jordan, but no significant difference was observed in these odds between households in South Jordan and those in North Jordan. With regard to condition, individuals with Arthritis had 70 % (CI:6–91) lower odds of care-seeking than those with hypertension; no significant differences in care-seeking rates were observed for other conditions.

**Table 2:** Characteristics by care-seeking decision and odds of care-seeking

	Overall	Sought Care	Did Not Seek Care	*p-value*	Odds of Care-Seeking*	
	(*N* = 1363)	(*N* = 1154)	(*N* = 209)		Crude OR (95 % CI)	Adjusted OR (95 % CI)
Household Head Educational Attainment (Highest Level Completed)						
None	17.8 %	20.3 %	2.9 %	0.180	Reference	Reference
Primary	27.3 %	26.6 %	31.4 %		***0.12 (0.02,0.92)***	***0.12 (0.02,0.87)***
Preparatory	30.6 %	30.4 %	31.4 %		0.14 (0.02,1.23)	0.20 (0.02,2.12)
Secondary or higher	24.4 %	22.7 %	34.3 %		***0.09 (0.01,0.65)***	***0.11 (0.02,0.78)***
Socioeconomic Quartile (based on monthly expenditures)						
Bottom	43.1 %	43.9 %	38.3 %	0.308	Reference	Reference
2nd	13.6 %	14.0 %	11.5 %		1.07 (0.60,1.90)	1.35 (0.25,7.17)
3rd	19.1 %	18.5 %	22.0 %		0.73 (0.47,1.15)	1.74 (0.29,10.60)
Top	24.2 %	23.5 %	28.2 %		0.72 (0.48,1.10)	0.94 (0.24,3.74)
Crowding (5+ / sleeping room)	13.2 %	12.4 %	17.7 %	0.085	0.66 (0.41,1.06)	1.95 (0.33,11.65)
Registered with UNHCR**	94.8 %	94.9 %	94.3 %	0.739	1.13 (0.55,2.34)	0.75 (0.08,7.34)
Assistance Received***						
Cash	29.0 %	29.5 %	26.3 %	0.436	1.17 (0.79,1.74)	1.33 (0.44,4.02)
Voucher	92.1 %	92.6 %	89.0 %	0.188	1.56 (0.80,3.02)	1.97 (0.52,7.43)
Non-food items	14.2 %	14.8 %	10.5 %	0.177	1.48 (0.83,2.62)	***13.25 (1.74,101.24)***
Year of Arrival in Jordan						
2011–2012	43.9 %	44.7 %	39.2 %	0.256	Reference	Reference
2012–2014	56.1 %	55.3 %	60.8 %		0.80 (0.54,1.18)	1.59 (0.46,5.53)
Region						
North	50.6 %	53.1 %	36.4 %	***0.004***	Reference	Reference
Central	45.1 %	42.4 %	60.3 %		***0.48 (0.31,0.74)***	***0.32 (0.10,0.99)***
South	4.3 %	4.5 %	3.3 %		0.92 (0.27,3.14)	0.44 (0.10,1.90)
Condition						
Hypertension	29.9 %	31.0 %	23.9 %	***<0.001***	Reference	Reference
Cardiovascular Disease	13.9 %	14.0 %	13.4 %		0.81 (0.53,1.23)	1.62 (0.51,5.20)
Diabetes	18.3 %	18.9 %	15.3 %		0.95 (0.62,1.46)	2.91 (0.87,9.79)
Chronic Pulmonary Disease	15.6 %	16.2 %	12.4 %		1.00 (0.65,1.56)	1.48 (0.35,6.33)
Arthritis	22.2 %	19.8 %	34.9 %		***0.44 (0.30,0.64)***	***0.30 (0.09,0.94)***

*Bold indicates statistically significant (*p* < 0.05) findings

**“Registered” individuals include those currently registered or awaiting registration

***Analyzed as dichotomous variables (assistance type received/not received)

## Discussion

An estimated 80 % of deaths worldwide are linked to NCDs [20]. These conditions are a particular concern among middle-income populations affected by conflict, which are likely to be older and have high NCD burdens, and require long-term treatment that is often resource intensive [20, 21]. There is growing recognition that NCDs represent an emerging challenge in humanitarian response, especially among urban refugee populations. In these settings, the international community and host country governments face numerous challenges in meeting refugee health needs. Management of NCDs is costly to donor agencies, depleting the limited resources available for refugee health care [22]. The caseload of Syrian refugees with NCDs in Jordan is but one example of an evolving trend among urban refugees [1].

## Prevalence of chronic health conditions

In Jordan, NCDs are estimated to account for 76 % of total deaths and the probability of dying from one of the four major NCDs between 30 and 70 years of age is 20 % [23]. Projected 2015 prevalence rates for NCDs among Jordanians were approximately 15 % for hypertension and 8 % for diabetes [24]. NCDs were estimated to account for 46 % of all deaths in Syria in 2010 and mean body mass index and mean fasting blood sugar were increasing in both males and females [25]. There are few reliable prevalence estimates of specific NCDs in Syria and neighboring countries; however, a 2008 study of adult residents of Aleppo estimated a 5.4 % prevalence of cardiovascular disease and additional published estimates from Syria include a 6.1 % prevalence of chronic respiratory conditions and 20.5 % diabetes prevalence [26–28]. This compares to Syrian refugee reported prevalence estimates in this study of 10.7 % for hypertension and 7.1 % arthritis, which were the two most common NCDs reported. A similar household survey conducted in Jordan in 2008 found prevalence among Iraqi refugees settled in Jordan similar to the results of this study as follows: hypertension, 19.6 %; diabetes, 9.1 %; cardiovascular disease, 6.7 %; lung/respiratory disease, 3.1 %; and musculoskeletal conditions (not restricted to arthritis), 18.5 % [29]. Similar care-seeking behaviors and barriers were observed among Iraqi refugees in Jordan with 85.8 % reported seeking care the last time it was needed and cost being the most commonly reported barrier to care (among 35 % of Iraqi refugees surveyed) [29]. While undeniable differences exist between the Syrian refugee and Iraqi refugee populations, commonalities in their settlement in Jordan and pre-crisis disease burden warrant a comparison of prevalence estimates for the two populations. In total, 43.4 % of Syrian refugee households reported that one or more household members had been previously diagnosed with one of the five conditions included in the survey. This compares to 45.7 % of Syrian refugee households reporting a member with a chronic health condition in a recent UNHCR survey [17].

When applied to refugee population data reported by UNHCR, the caseload of Syrian refugees with NCDs in Jordan was estimated as follows: hypertension 32,575; arthritis 21,534; diabetes 18,675; cardiovascular disease 12,562; and chronic respiratory conditions, 8699. It is likely that cardiovascular disease is considerably under diagnosed among the study population given the prevalence rates of reported hypertension, which at over 50 % in the 40–59 year group, is of great concern if substantiated by clinical assessment. This, with diabetes, portends a major burden of cardiovascular disease in the coming years for this population. If current displacement is protracted, this impending burden could entail substantial medical costs to UNHCR and the Government of Jordan, as well as increased utilization of referral hospitals by refugees.

### Care-seeking for chronic health conditions

Refugee care-seeking was high for all NCDs, with 84.7 % of those reporting an NCD had sought care in Jordan; the lowest care-seeking rates were observed among those with arthritis at 75.8 %. Across all conditions, the majority who elected not to seek care reported cost as the reason. This finding is aligned with other studies that report that cost is the main reason why refugees do not seek care for NCDs [17, 30–33]. Secondary and tertiary health care, when available to urban refugees, is usually expensive [1], in part because the cost of services is not subsidized, as is sometimes the case with primary care. However, in the case of Syrian refugees in Jordan approximately 70 % of those with an NCD accessed care for the condition within the 3 months preceding the survey and this figure increases to nearly 80 % for the 6 months preceding the survey. The high care-seeking rates observed in this study are aligned with data from a previous study on reported difficulties in care-seeking for NCDs in Jordan, in which 76 % of urban Syrian refugees reported experiencing no difficulties in attaining care [17]. One potential explanation is that the cost of NCD is not prohibitive for many refugee households. However, it may not be a priority given limited available funds for discretionary spending, and given recent decreases in humanitarian assistance, it is likely that cost will become an increasingly important barrier to care-seeking. Interestingly there was no association between socioeconomic status and care-seeking, which may be a reflection of successful targeting of assistance to the most vulnerable and/or the availability of low cost services in the public and NGO/charity sectors. Given the protracted nature of the crisis, the high costs of providing NCD care, the ability to prevent complications and associated costs if NCDs are controlled, and the large caseload of Syrian refugees with NCDs, implications for the Jordanian health system are immense. While the Jordanian Government and the international community continue to invest substantially in heath service provision for refugees, these efforts are falling short; as of April 2015, there was an 84 % shortfall in funds requested in the 2015 humanitarian appeal [8]. As a result of funding limitations and the high caseload, out-of-pocket payments are increasingly being used to help cover the costs of health service provisions. In late 2014, user fees for refugees increased which suggests that more refugees will face challenges in accessing basic services, particularly secondary and tertiary care, which are often very costly.

Other characteristics that were significantly associated with care-seeking for chronic conditions multivariate models were household head educational attainment and region. There was an inverse relationship between care-seeking and household head educational attainment, which was unexpected and could not be fully explained because the study lacked a qualitative component; one potential explanation is lower burden of disease among households with higher educational attainment. Households in which members had formal diagnoses of one or two NCDs also had higher levels of educational attainment whereas half of households with five or more NCD diagnoses among household members reported no formal education completed and only 25 % completed secondary or higher education. Another possible explanation is better management of conditions within this sub-group, which could result in care being received less often. Populations in the North region were more likely to seek care for NCDs than those in Central and South Jordan. There were no statistically significant differences in reasons for not seeking care by region, however, it is possible that refugees in the North are more aware of services because that is where the majority of the Syrian refugee population resides and many humanitarian response activities are focused on this region.

#### Limitations

With respect to sampling, reliance on UNHCR registration data may have resulted in sampling bias if the geographic distribution of registered and unregistered households differed. The within cluster referral process also presents the potential for bias, as respondents may not have always referred to the nearest household and instead to friends, which could have resulted in bias, though referral procedures and small clusters size may have attenuated within-cluster similarities and the associated design effect. Replacement sampling, which was done for logistical purposes, also could contribute to bias if there are systematic differences between households where no one was at home compared with those interviewed. Additionally, interviews were conducted by Jordanians which could have resulted in a higher refusal rate, hesitance or influence on the part of Syrian refugees in responding to certain questions than if interviews had been conducted by Syrians. With regard to non-communicable diseases, the fact that respondents self-reported the prevalence of diagnosed conditions in their household could mean that certain conditions were over- or under-reported. Some respondents may not have been aware of another household member’s diagnosis, or might not have thought that a household member’s diagnosis matched the disease category described by the interviewer. Where possible, efforts were made to involve adults other than the primary respondent in the interview if questions pertained to them to improve the accuracy of findings. With respect to socioeconomic status, it is possible that refugees were either unable or elected not to accurately report household income and expenditures; this could have affected our ability to understand and characterize the relationship between socioeconomic status and care-seeking. Finally, although this study captured data on whether or not household members sought care and did not focus on quality of care, examining the quality of the health services that care-seekers received is an important topic for future studies. It may be the case that although many Syrian refugees are seeking care, the care they are receiving is inadequate and leads to equally poor health outcomes.

## Conclusions

Non-communicable diseases are of increasing importance in urban refugee populations. The Middle East has a high NCD burden, and Syrian refugees are no exception. More than half (50.3 %) of Syrian refugee households had a member with a NCD and the caseload of Syrian refugees in Jordan with NCDs was estimated at more than 90,000. While NCD care-seeking was relatively good and 84.7 % of Syrian refugees with NCDs sought care, there is a significant minority that did not receive care and cost was the primary barrier to care-seeking. Identifying the means to maintain and improve access to NCD care for Syrian refugees in Jordan is essential. As funding limitations persist and the economic situation of refugees remains tenuous, focused effort to ensure that refugees are able to seek and afford care NCD care is critical. Investing in access to NCD services will not only benefit refugees, it will also reduce the long-term burden and costs of NCD care for the Jordanian health system.

## Footnotes

1 Includes heart failure, angina, arrhythmias [irregular heartbeats], a previous heart attack, or previous stroke

2 Includes asthma, chronic bronchitis, emphysema, and chronic obstructive pulmonary disease

3 Reported age-specific prevalence is weighted by the number of household members in each age group. Adult prevalence is weighted by the number of household members over 17 years old reported by respondents.
